# Extracting multiple interacting root systems using X‐ray microcomputed tomography

**DOI:** 10.1111/tpj.13047

**Published:** 2015-12-07

**Authors:** Stefan Mairhofer, Craig J. Sturrock, Malcolm J. Bennett, Sacha J. Mooney, Tony P. Pridmore

**Affiliations:** ^1^ School of Computer ScienceUniversity of NottinghamJubilee CampusNottinghamNG8 1BBUK; ^2^ Centre for Plant Integrative BiologyUniversity of NottinghamSutton Bonington CampusLoughboroughLE12 5RDUK; ^3^ School of BiosciencesUniversity of NottinghamSutton Bonington CampusLoughboroughLE12 5RDUK

**Keywords:** X‐ray computed tomography, root system interaction, multiple target tracking, technical advance

## Abstract

Root system interactions and competition for resources are active areas of research that contribute to our understanding of how roots perceive and react to environmental conditions. Recent research has shown this complex suite of processes can now be observed in a natural environment (i.e. soil) through the use of X‐ray microcomputed tomography (μCT), which allows non‐destructive analysis of plant root systems. Due to their similar X‐ray attenuation coefficients and densities, the roots of different plants appear as similar greyscale intensity values in μCT image data. Unless they are manually and carefully traced, it has not previously been possible to automatically label and separate different root systems grown in the same soil environment. We present a technique, based on a visual tracking approach, which exploits knowledge of the shape of root cross‐sections to automatically recover from X‐ray μCT data three‐dimensional descriptions of multiple, interacting root architectures growing in soil. The method was evaluated on both simulated root data and real images of two interacting winter wheat Cordiale (*Triticumaestivum* L.) plants grown in a single soil column, demonstrating that it is possible to automatically segment different root systems from within the same soil sample. This work supports the automatic exploration of supportive and competitive foraging behaviour of plant root systems in natural soil environments.

## Significance Statement

Imaging roots in their natural soil environment is important for understanding their growth, development and resource competition. Here we describe how interacting root systems can be separated and visualised using X‐ray micro Computed Tomography images. This allows in‐situ analysis of sets of root systems, which is of particular interest in intercrop cultivation.

## Introduction

Plants use their root systems to explore the heterogeneous and complex soil environment for water and nutrient sources which, in the field, are shared with other, neighbouring plants. Each plant must compete for its survival, especially under stressful conditions, when these resources are limited. Root system interaction and belowground competition in plant communities are subjects of wide interest (Mahall and Callaway, 1992; Casper and Jackson, 1997; Rubio *et al*., 2001; Maina *et al*., 2002). Root competition is considered a negative aspect of root interaction, in which plants can limit each other's growth. However, root interaction can also have positive effects, for example by simultaneously decreasing the availability of one resource while increasing the availability of another or by influencing the composition of the bacterial flora in the rhizosphere, which may affect the availability of nutrients to neighbouring plants (Schenk, 2006). These interactions are of particular concern for intercrop cultivation, which is of significant and increasing interest at present (Brooker *et al*., 2014); here the aim is to find the optimal combination of plants for a certain field environment. Planting strategy can have a large effect on crop yield (Mead and Willey, 1980; Willey, 1985; Anil *et al*., 1998). It is commonly believed that root systems have the ability to sense neighbouring plants, though the process is complicated and not yet fully understood due to an inability to visualise *in situ* root behaviour. Some studies have shown that roots of the same genotype within a species have a tendency to grow towards each other, while the roots of another genotype within the same species avoid sharing the same area (Fang *et al*., 2013). However, this mechanism appears to be ignored if plants are grown in resource‐limited environments (Caffaro *et al*., 2013). To fully comprehend the degree to which root interaction affects root development, it is essential to observe and study root behaviour in the natural, heterogeneous soil environment (a previous limitation due to the opacity of soil). Non‐soil environments typically allow more rapid and widespread diffusion of chemical compounds, producing signalling patterns different from those that would be observed in natural soil cultivations (Chen *et al*., 2012).

Belowground competition between roots grown in soil is usually inferred by measuring the availability of resources in the soil, the presence or absence of roots in those areas and the rate of uptake of these resources or other measurable plant traits (Schenk, 2006). Gersani *et al*. (2001) designed a split‐root experiment in which plants either shared their root systems in two adjacent pots or were kept separated, with each plant limited to its own pot. Data were collected by destructive harvesting of the plants. Similar experimental designs were used by Maina *et al*. (2002) and O'Brien *et al*. (2005). Their studies investigated the ‘tragedy of the commons’ in which plants that compete below ground produce increased root biomass by sacrificing yield.

Non‐invasive observation and time‐series analysis of multiple plant root systems would provide further insights into coexistence and competition within plant communities by allowing a more comprehensive understanding of the conditions and stages when plants start reacting to their neighbours. Faget *et al*. (2013) combined magnetic resonance imaging (MRI) and positron emission tomography (PET) to image root systems of two maize plants. Radioactive ^11^CO_2_ was introduced into the shoot of one of the plants and it was possible to measure its transport through the root systems using PET. This method could potentially be used to distinguish interacting plants. X‐ray microcomputed tomography (μCT) has been well described as a popular alternative to MRI for non‐destructive analysis of plant roots grown in soil (e.g. Mooney *et al*., 2012). X‐ray μCT allows the observation of roots in soil by measuring the attenuation of ionising radiation passing through a sample of interest. The degree of X‐ray attenuation depends on the density of the material, the number of photons transmitted through the object being inversely proportional to its density. Though image segmentation (sometimes known as phase separation) is challenging, differences in density allow roots to be distinguished from their surrounding complex soil environment. As all the root material in the sample is likely to cause comparable X‐ray attenuation, its greyscale values in the resulting image data are typically very similar. Therefore if multiple plants are grown within the same soil column, it will not be possible to distinguish and assign their roots to the correct plant of origin using conventional image analysis approaches (e.g. simply by applying threshold tools), which might explain why this approach has not been previously developed. While a slight difference in density might be observable among certain plant species, it is unlikely to assist image segmentation and there will be no/little difference if plants of the same variety are examined.

**Figure 1:**
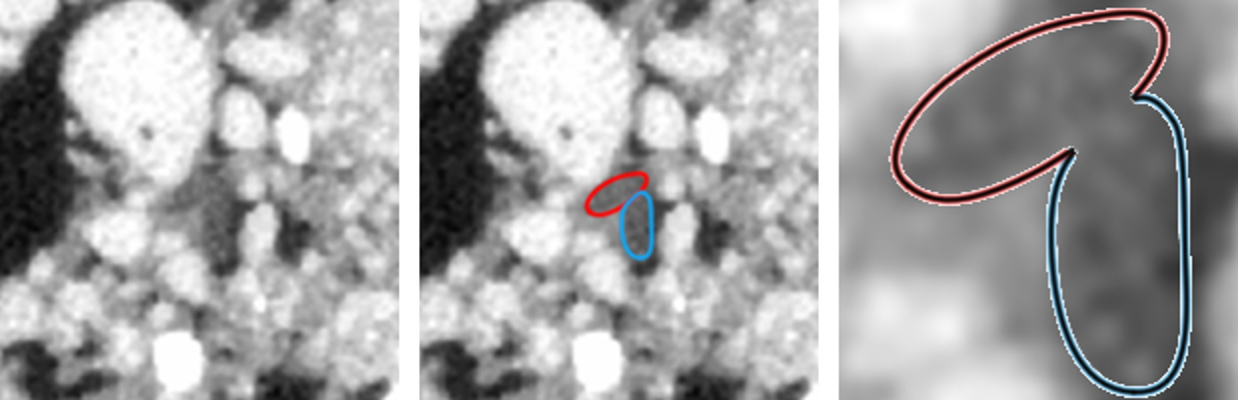
Two interacting roots. Two interacting roots originating from different root systems: the boundary between them is unidentifiable: (a) microcomputed tomography image; (b) root sections marked; (c) zoom in to the root objects.

Recently RooTrak, a software tool capable of recovering plant root systems from X‐ray μCT image data, has been presented (Mairhofer *et al*., 2012, 2013). RooTrak uses a visual tracking framework based on the application of the level set method (Sethian, 1999) and the Jensen–Shannon divergence (Lin, 1991). The X‐ray μCT data are viewed as a sequence of cross‐sectional images through which root objects are tracked. Each root cross‐section is thought of as a moving target belonging to and emerging from a root system. This means that if a new target appears from an emerging lateral root, it will be considered an individual object to be tracked, while at the same time being associated with the root system from which it originated. This makes it straightforward to apply a separate group of trackers to the root system of each individual plant when multiple plants coexist. Complications arise, however, when root objects from different plants collide/touch, which happens when roots come into contact with each other. The restricted set of greyscale values arising from root material means that the boundary between these objects has low contrast at best and is potentially non‐existent, causing root sections to visually merge into a single object, as shown in Figure 1. The problem of ‘coalescence’ of interacting targets is a widespread feature of multiple target tracking and an active research topic in computer vision (Milan *et al*., 2013). All visual trackers rely heavily on an appearance model of some form; similar targets will always be tracked with a similar, and often identical, model. When targets interact, each tracker will tend to lock on the target that best fits the model. This can result in trackers swapping targets or trackers following the same target while losing track of others. In the case of root material recovery, this leads to root cross‐sections being associated with incorrect root systems.

In this work we present a mechanism based on the root recovery method presented by Mairhofer *et al*. (2012) but with the significant added ability to extract multiple interacting root systems. The aim is to develop an X‐ray μCT image analysis tool that can aid understanding of how plants interact and communicate through their root systems, supporting new research efforts into the biophysical behaviour of roots that is important, for example, for optimising mixed/intercropping systems. In what follows, we give a detailed description of the proposed extraction mechanism (Data S1 online), which is applied to both simulated and real plant X‐ray μCT data.

## Results

### Extended root extraction mechanism for multiple plants

Extracting multiple root systems from μCT data is a very similar process to recovering a single root system, until two or more root systems interact. The original RooTrak algorithm (Mairhofer *et al*., 2012) tracks multiple root cross‐sections, allowing them to split and merge without restriction. When more than one root system is present, unconstrained merging of root cross‐sections is problematic, as it fuses together root material from different plants. To overcome the critical phase in which roots interact, our proposed solution relies on utilising measures of the shape of the root cross‐sections concerned. Shape information is used to refine and control boundary extraction by the level set method and thus helps to improve the reliability of the recovery process by locking each tracker to its target, preventing merging of material from different plants. The following elements are key to the proposed extraction mechanism: (i) detection of root object collisions; (ii) root shape registration, and (iii) refinement of the level set boundary evolution.

Given the approach in Mairhofer *et al*. (2012), the extraction of multiple root systems requires tracking of cross‐sections belonging to multiple root systems. This in turn requires multiple instantiations of the level set technique to be active together. The level set method was initially developed to evolve the interface of a single front that is defined by the transition from negative values inside to positive values outside the object boundary (Osher and Sethian,^1988^; Sethian, 1999). This has been extended by simulating the flow of two‐phase fluids (Sussman *et al*., 1994, 1999) and the complex interaction of more than two fluids (Merriman *et al*., 1994; Sethian, 1994; Losasso *et al*., 2006). In the work reported here we adapt the solution defined in Sethian (1994), where multiple level set functions are evolved simultaneously. This and similar approaches have been established as a popular technique in computer vision, used for example in the segmentation of greyscale and colour images into multiple distinguishable regions (Vese and Chan, 2002). The method enjoys the advantages of simplicity and efficiency, but lacks the high precision required in many physics‐based applications (Losasso *et al*., 2006). The key idea is that the interface of a level set function can only be moved to a new position if none of the other level set functions already have that particular location included in their interface. If the location is already occupied, the sign of the updated value is switched from a negative value (inside) to a positive value (outside). Ignoring this rule for one level set function, however, allows one interface to enter another's boundary which, as a consequence, will be pushed back. The approach has the additional advantage of providing an easy way of identifying collisions between objects, by determining the number of level set functions that share negative values for a certain location in the image data.

As RooTrak proceeds in a top‐down approach through the μCT image stack, considering each cross‐sectional image in turn, the level set interface adapts to identify the new location and form of the target, tracking root branches. In the original algorithm the region bounded by the interface is simply output, providing a description of the root material visible in that image. In the technique presented here the outline of the interface is recorded as a set of points. This allows the iterative closest point (ICP) algorithm (Besl and McKay, 1992) to be applied. The ICP algorithm is a technique that takes a set of points and aligns them to another point cloud, aiming to find the rotation and translation matrix that minimises the distance between corresponding pairs of points. Our algorithm therefore keeps note of the shape of each root object that is tracked through the image stack and is able to recall the outline of an object's shape at any subsequent stage of tracking, using ICP to align it to the current position of the level set interface.

Upon occurrence of a collision between root objects from different plants, the detection routine triggers the proposed extraction mechanism for interacting root objects, which will run as long as the targets remain in contact. The mechanism starts by considering the most recently stored description of the shape of the interface, recorded before the collision. The same shape information is used throughout the entire interaction phase, i.e. no further shape information is recorded until the level sets are once again clearly separated. It is assumed that the shape of a root cross‐section is approximately constant during the period of contact. We accept that this assumption may not always hold; however, we hypothesise that it is reasonable to believe it will be true in the majority of cases, since the number of cross‐sections at which a root bends (and so its cross‐section changes shape) is low in comparison with the number of slices through which the root follows a smooth path. When a bend occurs, we hypothesise here that it is also more likely that the root will bend away from neighbouring roots and so the tracked root object will lose contact with any neighbouring roots and hence leave the critical collision zone of the corresponding image. In order to violate our hypothesis, two neighbouring roots would have to turn away at the same time with the same angle and direction. In that case the collision phase is still ongoing, while the shape of the roots would drastically change and therefore become invalid. Such a scenario has not been observed in any of the test data sets used in this work.

**Figure 2:**
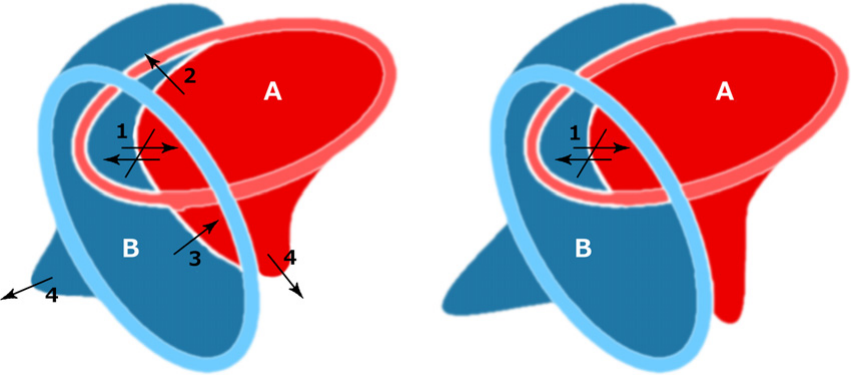
Interacting level set functions. Interacting level set functions A and B (a) before and (b) after evolution: (i) neither A nor B can be pushed; (ii) A pushes the interface of B; (iii) A is pushed by the interface of B; (iv) A and B evolve normally.

When tracking through a collision we apply the ICP algorithm to co‐register the pre‐stored shape to the current interface of the level set. This leaves each point on the interface (labelled A in Figure 2) in one of two possible states: it is either outside or inside its aligned region. If an interface location is outside the region predicted by the ICP algorithm, the front, and so the root cross‐section, has grown. The level set method has no information that indicates whether or not this is the correct interpretation, and so the level set function evolves as normal. If a point now lies inside the region predicted by its recent shape it is possible, indeed likely, that it is being pushed inwards by the colliding object. It is therefore given the ability to push back the boundary of the other level set and reclaim its previous shape. The same strategy is adopted by the other, interacting level set function (labelled B in Figure 2), which leads to the following scenarios (Figure 2):

- A and B evolve normally (4).
- A pushes the interface of B (2).
- A is pushed by the interface of B (3).
- Neither A nor B can be pushed (1).

The combined effect is that each level set attempts to maintain its previous shape, reducing the likelihood that either level set (and so root system) will extend into and be confused with another. Shape information and the ICP mechanism effectively add a further constraint to the root recovery algorithm during the interactions of root objects, in addition to the previous assumption of smoothly varying density distributions. If there is no collision between root objects the shape constraint is not active, and therefore results are obtained as with the original RooTrak. The presented mechanism is also applicable to upward‐oriented roots, following the approach presented in Mairhofer *et al*. (2013). Roots that are inclined by even a slight angle are usually easier to deal with than horizontally grown roots, as there is likely to be some variation in position or appearance, and because they are processed by the tracker in a step‐wise fashion. Horizontal roots are not impossible to cope with, but there are more possibilities for the tracker to be misguided. Even if a root grows horizontally, it will span a few images, and therefore give the tracker the opportunity to recover it by alternating the direction in which target objects are sought (Mairhofer *et al*., 2013).

### Simulated data

To test and demonstrate the impact of the proposed mechanism, compared with the original root system recovery method, a set of artificially generated image sequences were created (Mairhofer *et al*., 2014). Root objects were represented by circles of radius 20 pixels that were moved a distance of 6 pixels between images. The direction of movement was randomly selected within an angle between −16° and +16° to simulate a random path. The circles were bounced off the image boundaries to keep the root sections within the scene and to ensure they were trackable throughout the entire sequence. Image size was restricted to 320 pixels × 320 pixels and a total of 500 images were generated. Two roots were simulated in each image stack, each following a random path, which due to the limited space caused them to interact at several points. Independent simulations were performed to generate a total of 12 different image stacks.

### Plant root systems

After running the proposed method on an artificially generated dataset, it was then applied to real data acquired from actual plant samples. Five columns were prepared, each containing two winter wheat Cordiale (*Triticumaestivum* L.) seeds. The seeds were planted 10–15 mm apart after having been left to germinate for 2 days on wet filter paper in a Petri dish shielded from light. The columns, 30 mm in diameter, were filled with a Newport series loamy sand soil. The soil was air dried and sieved to <2 mm. The columns were placed in an environmentally controlled growth room with 16‐h/8‐h light cycle at a temperature of 23°C/18°C, and left there for 10 days before the plants were examined. The samples were scanned using a Nanotom (Phoenix X‐ray/GE Measurement and Control Systems, http://www.phoenix-xray.com/) X‐ray μCT scanner. The scan was performed at 120 keV and 110 μA, taking 1440 projections with an exposure time of 750 ms, using signal averaging of three and one skips per projection. The samples were placed 134 mm away from the X‐ray gun, resulting in a volume with a resolution of 22.33 μm^3^ voxel size. The X‐rays were filtered through a 0.1‐mm copper plate. The total scan time for each sample was 77 min. Note that using a single plant species means that all the root material present in the image data will generate intensity values drawn from the same distribution. A tracker is initialised by manually setting seed points at the beginning of a root system. The user specifies which tracker each seed point belongs to, which allows the selection of multiple seed points for a single tracker. This is useful if the top of the image data is missing and the tracking has to be started from individual nodal roots.

### RooTrak results

**Figure 3:**
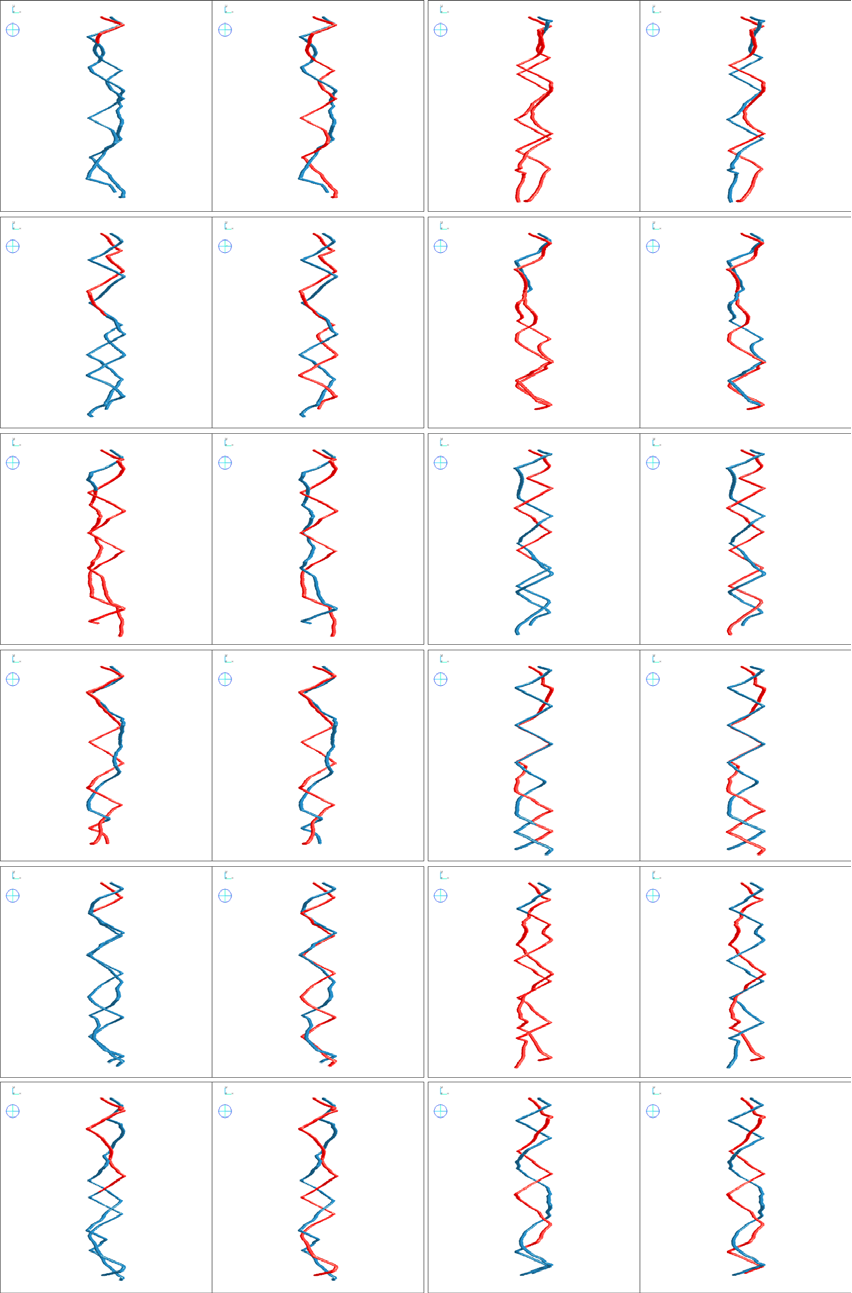
Root descriptions are extracted from interacting root simulations. Root descriptions are extracted from interacting root simulations by (left) the original RooTrak and (right) the proposed mechanism with an active shape constraint, for which objects were correctly labelled throughout all the interactions.

**Figure 4:**
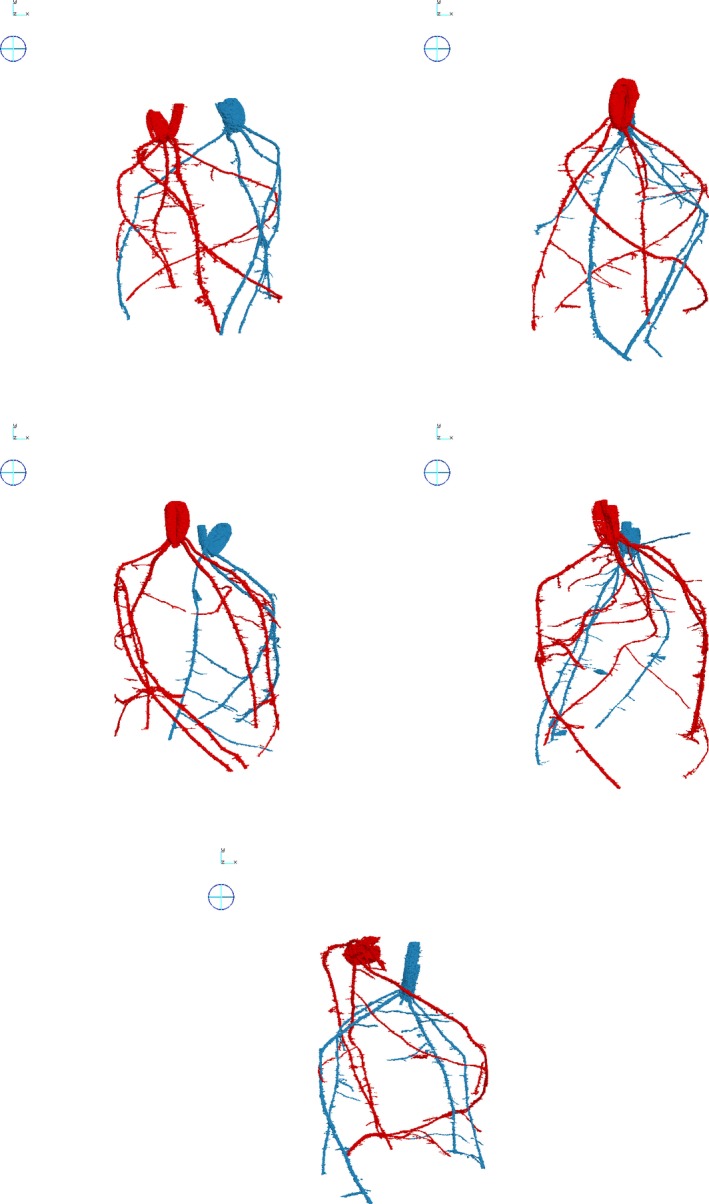
Recovery of root system descriptions. Root system descriptions recovered from real images of two interacting wheat plants from five prepared and scanned samples. The growing conditions for all five samples were the same. Multiple samples were used: because of the complexity of plant roots a wide range of interactions is possible.

**Figure 5:**
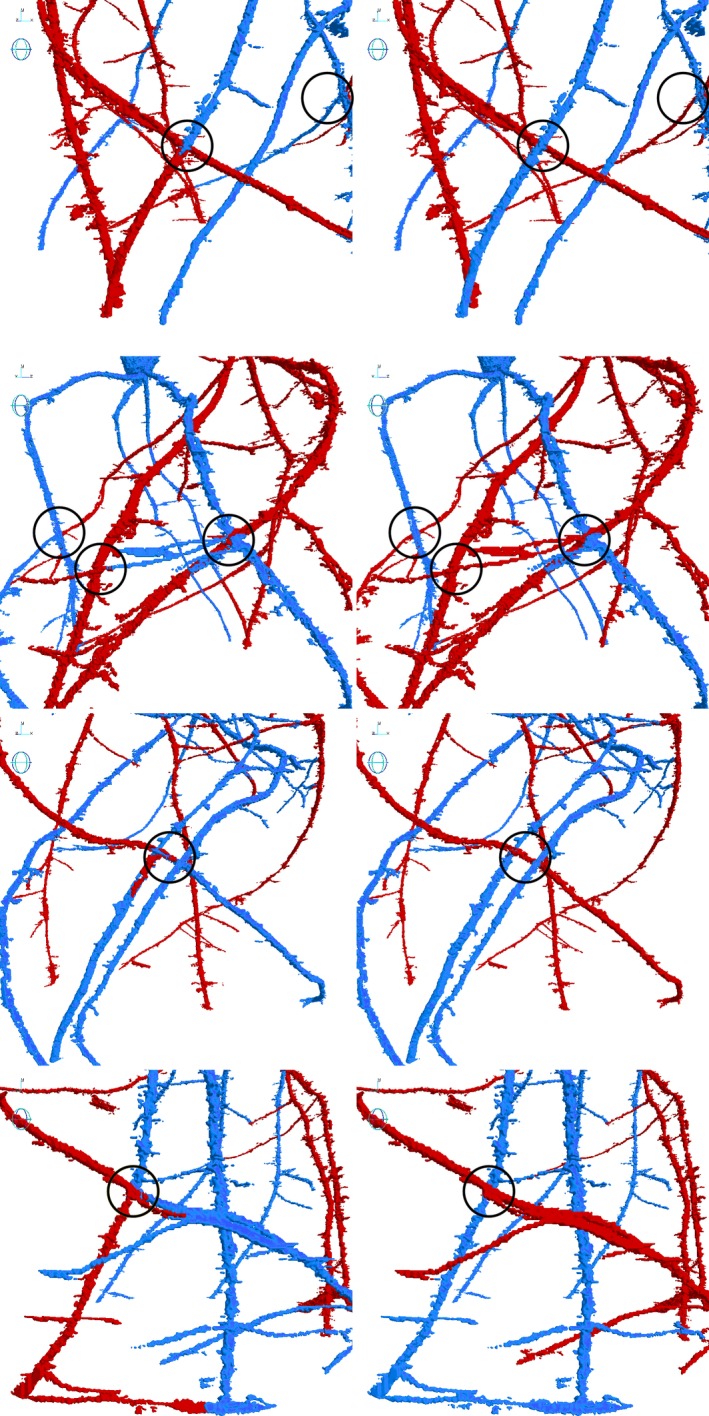
Extraction and enlargement of root descriptions. Root descriptions extracted and enlarged for comparison: (left) the original RooTrak; (right) the proposed mechanism.

**Figure 6:**
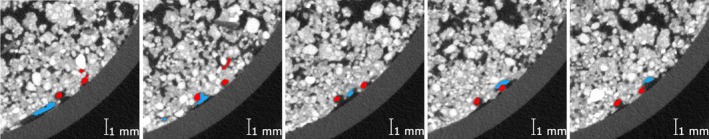
Real image sequence. Real image sequence showing root objects interacting with each other, while being labelled as separate plants.

The root system descriptions recovered from the experiment performed on the simulated roots are shown in Figure 3. On the left side of each pair is the result obtained using the original extraction method (Mairhofer *et al*., 2012), while on the right side the proposed mechanism was activated each time it was triggered by two interacting targets. In samples 1–12 there were a total of three, two, two, four, one, one, two, two, three, three, two and one interactions, respectively; interactions were of varying duration with varying degrees of overlap between objects. For all the samples, the tracker correctly labelled the objects during collision. Figure 4 shows the results of the experiment performed on real images of the root systems of two interacting wheat plants; each root system is rendered in a different colour. Figure 5 shows the same root systems, but from viewpoints closer to interacting roots, illustrating the difference between the original version of RooTrak and the proposed mechanism to deal with root object collisions. Figure 6 shows a sequence of cross‐sectional images in which the roots of the two interacting wheat plants were identified, while at the same time kept separate and assigned to the correct originating plant by to the mechanism proposed here. The time needed to recover the root systems from the CT images depends on the number of data and the number of root objects being tracked. The root systems in this experiment were extracted within 4–5 h on a Windows 7 64‐bit desktop PC, Intel Core i7‐3820 3.60 GHz, 32.0 GB RAM. A GPU implementation is under construction which is expected to reduce processing times significantly. The diameters of the roots extracted from the data varied from 15 to 30 pixels. These values are only an indication of the root objects being recovered in the context of this experiment, and do not represent the minimum target size for which the algorithm is applicable. From a computational point of view, an object of radius 1 pixel would be sufficient to serve as a target, but not very realistic in an operational context, where there is variation in the appearance and location of the object.

## Discussion

The root descriptions extracted from the simulated data show the value added by the proposed mechanism. They also demonstrate that when multiple roots are present, and without any additional constraints, the assignment of an object (root segment) to its originating target (plant) can be difficult and unreliable using the original version of RooTrak. Although the underlying aim of separating target (root material) from background (soil) was successfully achieved in both cases, it can be observed how easily targets were passed between trackers, making it impossible to tell whether a given root segment really belongs to the plant to which its tracker was initialised. While root interaction does not always lead to a swap or loss of tracking, it remains something that needs to be considered when extracting multiple roots, particularly if plants are densely planted. The random paths assigned to the artificial roots generated interactions from various directions. Root objects interacted by coming together from opposite directions as well as by following the same direction, with one catching up with the other. With the proposed mechanism active, the method performed much better at keeping track of the correct target, and as such increases confidence in its ability to distinguish objects with a similar appearance.

Using the mechanism proposed here for the recovery of multiple root systems, we extracted the root systems of two interacting wheat plants. While the shape constraint increases the likelihood of roots being assigned to the correct root system, it does not guarantee perfect separation between them. There are still cases in which incorrect assignments are made. One such scenario arises when the trackers of two different root systems pick up a single U‐shaped root at different ends, following the target until they eventually meet in the middle of the root. Additional information on branching angles might help in identifying whether a root is more likely to belong to one root system or the other, and could therefore be used to re‐label incorrectly assigned root branches to their originating plant.

**Figure 7:**
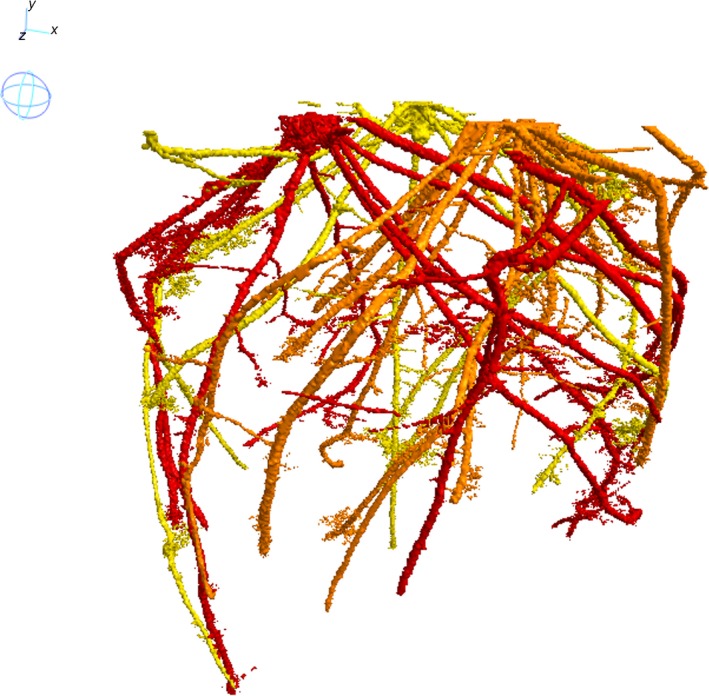
Recovered root systems of three interacting wheat plants. Recovered root systems of three interacting wheat plants: each root system is labelled and assigned to its plant of origin.

Importantly, the method proposed here is not limited to recovering and separating two plants, but can work on a number of root systems that interact with each other. Figure 7, for instance, shows the recovered root systems of a sample in which three wheat plants share the same soil environment. This method could also be extended to mixtures of plant species, which is especially important at present given a renewed interest in the potential soil‐structuring and compaction‐alleviating capabilities of competing plant species. Chen and Weil (2011) showed that maize grown under compacted conditions benefited from a pre‐cover crop such as forage radish (*Raphanus sativus*) and rapeseed (*Brassica napus*) as their root systems can effectively ‘bio‐drill’ the soil to enhance porosity for future plants. Potentially, mixes of species could also reduce nutrient losses, improve drainage, and minimise soil erosion. In addition, Postma and Lynch (2012) have shown there can be great complementarity for combinations of plant species, such as enhanced N uptake and biomass production for maize/bean/squash polycultures. Multicropping may also offer potential advantages in terms of pest and disease control and soil health (Lithourgidis *et al*., 2011). Recently there have been numerous examples from around the world where combinations of plants grown together, typically including radish, oats, rye, mustard and sunflower, have improved subsequent soil quality and plant growth. However, these are largely unpublished studies, possibly because a suitable method for exploring root interactions *in situ* has not existed until now. The method proposed here may provide a solution. A further advantage of using X‐ray μCT that is not considered here is that CT imaging also provides information concerning the soil structure and porosity.

While the wheat pilot study demonstrates the ability of the method to separate the root systems of different plants, the acquired data do not allow any conclusions to be drawn regarding competition between interacting plants. The columns chosen for the experiment were not wide enough to prevent roots from reaching the boundary and so being diverted and restricted in their development. It has already been shown by Schenk (2006) that pot size has a significant influence on root development, in particular when it comes to the study of root competition. Also most interactions occurred at the boundary surface, and so are likely to have arisen from limited space rather than signalling between plants. Limiting the space, however, increases the chance of interaction, which was significant for this work. Growing multiple plants in the same soil environment requires larger columns in order to prevent the influence of external factors, which in turn requires an X‐ray CT scanner that allows bigger samples to be scanned. While such systems exist, it is worth noting that for X‐ray CT a larger sample size usually leads to coarser resolution which would have a negative impact on the detection of roots, or at the least lead to a very significant increase in scan time, generally not considered ideal in phenotyping efforts, using smaller regions of interest that can be combined to create an image dataset of a larger volume. From this and previous work we found a resolution of 25 μm^3^ and below to be suitable for the detection of wheat root systems. However, results can vary depending on image data quality. The analysis of lower‐resolution image data from larger columns is a subject for future research efforts.

## Conclusion

A technique has been presented, based on the RooTrak method of recovering descriptions of root system architecture from X‐ray μCT images of roots grown in soil, that allows the roots of multiple plants to be separated. The proposed mechanism was tested in an experiment on simulated roots as well as real images showing two interacting wheat plants grown in the same soil environment. The results clearly show that it is now possible to extract multiple interacting root systems, a significant advance over the previous extraction process in which no shape constraint was applied upon collision. While no guarantee can be given that root objects are associated to the correct plant, the additional operation adds a higher degree of certainty. Only by explicit reasoning about the structure of the root system architectures of particular species would it be possible to increase confidence in assigning root objects to the right plant. However, this is a very challenging task, as root system architectures vary considerably with species and environment. Nonetheless, we believe that the extracted data allow us to obtain a good indication of the overall interaction between multiple root systems and now provides meaningful information for the study of interacting and competing plant root systems in natural soil environments.

## Experimental procedures

### Plant growth

Wheat Cordiale (*Triticumaestivum* L.) seeds were germinated in Petri dishes. After 2 days they were planted 10–15 mm apart in plastic columns filled with loamy sand soil sieved to <2 mm. All plants grew in environmentally controlled growth rooms with a 16‐h/8‐h light cycle at a temperature of 23°C/18°C and were scanned 10 days afterwards.

### Imaging

All μCT data were acquired at the University of Nottingham using a Nanotom (Phoenix X‐ray/GE Measurement and Control Systems) X‐ray scanner. The scanning resolution was 22.33 μm^3^, the voltage 120 keV and the current 110 μA. We took 1440 projections with an exposure time of 750 ms, using a signal averaging of three and one skips per projection. The samples were placed 134 mm away from the X‐ray gun. The X‐rays were filtered through a 0.1‐mm copper plate. The total scan time for each sample was 77 min.

## Supporting information


**Data S1.** Extracting multiple interacting root systems using X‐ray microcomputed tomography.
