# Supplementary material for: Extracting multiple interacting root systems using X‐ray microcomputed tomography
Source: Plant J. 2015 Dec 7;84(5):1034–43. doi: 10.1111/tpj.13047 (PMC4949686; doi:10.1111/tpj.13047)
Supplement: Supplementary file 1 — Data S1. Extracting multiple interacting root systems using X‐ray microcomputed tomography. [file TPJ-84-1034-s001.pdf]

Supplemental material: Extracting Multiple Interacting Root Systems using X-ray Micro Computed Tomography

Here we give a detailed description of the proposed extraction technique, beginning with the extraction of a single individual root system (i.e. assuming that all root cross-sections belong to the same plant). A collision detection mechanism is then added to identify the interaction of multiple targets, to which a shape constraint is imposed, allowing the extraction of multiple interacting plant root systems.

Object Boundary Detection

We adopt the level set framework (Sethian, 1999) to search for the boundaries of root cross-sections. We aim at finding the interface

$$C\left(t\right)=\left\{\left(x,y\right)\middle|\Phi_{x,y,t}=0\right\} \tag{1}$$

of a time-dependent function $\Phi_{x,y,t}$ that separates an object consisting of comparable intensity values from its heterogeneous background. The interface of $\Phi_{x,y,t}$ can be implicitly propagated by solving a partial differential equation

$$\frac{\partial\Phi_{x,y,t}}{\partial t}+F\left|\nabla\Phi_{x,y,t}\right|=0 \tag{2}$$

which can be approximated and rewritten using a finite forward difference scheme in time

$$\frac{\Phi^{t+1}_{x,y}-\Phi^{t}_{x,y}}{\Delta t}+F\left|\nabla_{x,y}\Phi^{t}_{x,y}\right|=0 \tag{3}$$

giving a general formulation of the time-discretized level set method, with F being a speed function that defines the motion of the front over time t. Assuming we have the greyscale intensity values of a known root object, we use a kernel density estimator to build a statistical probability density function, which we will refer to as our root appearance model p_m

$$p_m(x)=\frac{1}{nh}\sum_{i=0}^{n}K\left(\frac{x-x\left(i\right)}{h}\right) \tag{4}$$

where n is the number of data points, h the bandwidth and K a Gaussian smoothing kernel $K(x) = \frac{1}{\sqrt{2\pi}} e^{-\frac{1}{2}x^2}$. Using the Jensen-Shannon (JS) divergence (Lin, 1991) as given in Equation 5, we compute the distance between a probability density function p_f estimated around the interface of the level set function and our known root model p_m

$$JS(p_f, p_m) = H(w_1 p_f + w_2 p_m) - w_1 H(p_f) - w_2 H(p_m) \tag{5}$$

where H is the Shannon entropy function calculated as in Equation 6. w_1 and w_2 are two weighting parameters $w_1, w_2 \geq 0, w_1 + w_2 = 1$ used to balance the contribution of the two statistical probability density functions and useful for conditional probability studies where the weighting parameters represent prior probabilities. In our case, however, we set $w_1 = w_2 = 0.5$.

$$H(p) = -\sum_{i=0}^{n} p_i \, log_b \, (p_i) \tag{6}$$

The JS divergence is a non-negative and symmetric dissimilarity measure, bounded by $[0, log_b 2]$. Using a logarithm of base 2 results in a distance that is measured within $[0, 1]$, where 0 is considered a complete match between two probability density functions. Given the above definitions, we can now build them into a level set framework

$$\Phi_{x,y}^{t+1} = \Phi_{x,y}^{t} + \Delta t \left[- (\alpha) \left(JS_{\beta\vee} \nabla^{+} + JS_{\beta\wedge} \nabla^{-} \right) + (1 - \alpha) (\kappa) \right] \tag{7}$$

Multiple Interacting Objects

While tracking target objects through the image stack, their shape is noted and used to control appearance model updates. We can, therefore, easily recall an object's outline and store the most recent shape information seen before the interaction with other objects began. This information is kept until the interaction ceases. Let $U = \{\vec{u}_i | i = 1..N_u\}$ be a set of data points along the outline of a stored shape and $V = \{\vec{v}_i | i = 1..N_v\}$ be a set of data points along a level set's interface. The rotation matrix $\mathbf{R}$ and the translation matrix $\mathbf{T}$ are sought which minimise the root mean squared distance between U and V and therefore find the best alignment of the two point sets. This can be achieved using the iterative closest point (ICP) algorithm (Besl and McKay, 1992). By calculating the centre of mass $\vec{\mu_u}$ and $\vec{\mu_v}$ of the two point clouds, it is possible to determine the cross-covariance matrix $cov_{uv} =$

$\dfrac{1}{N_u}\sum_{i=1}^{N_u}[(\vec{u_i}-\vec{\mu_u})(\vec{v_i}-\vec{\mu_v})^\intercal]$ for U and V. Using the cyclic components $\vec{a}=(A_{23},A_{31},A_{12})$ of a matrix $\mathbf{A}=cov_{uv}-cov_{uv}^\intercal$ allows the definition of a 4×4 matrix $\mathbf{Q}$

$$\mathbf{Q}_{4\times 4}=\begin{pmatrix} tr(cov_{uv}) & \vec{a}^\intercal \\ \vec{a} & cov_{uv}+cov_{uv}^\intercal-tr(cov_{uv})\mathbf{I}_3 \end{pmatrix} \tag{8}$$

The eigenvector $\vec{r}=(q_1 \quad q_2 \quad q_3 \quad q_4)$ of the matrix $\mathbf{Q}$ with the maximum eigenvalue is used to define the rotation matrix $\mathbf{R}$

$$\mathbf{R}=\begin{pmatrix} q_1^2+q_2^2-q_3^2-q_4^2 & 2(q_2q_3-q_1q_4) & 2(q_2q_4+q_1q_3) & 0 \\ 2(q_2q_3+q_1q_4) & q_1^2+q_3^2-q_2^2-q_4^2 & 2(q_3q_4-q_1q_2) & 0 \\ 2(q_2q_4-q_1q_3) & 2(q_3q_4+q_1q_2) & q_1^2+q_4^2-q_2^2-q_3^2 & 0 \\ 0 & 0 & 0 & 1 \end{pmatrix} \tag{9}$$

The vector $\vec{t}=(\vec{\mu_v}-\mathbf{R}\vec{\mu_u})$ is used to define the translation matrix $\mathbf{T}$

$$\mathbf{T}=\begin{pmatrix} 1 & 0 & 0 & t_1 \\ 0 & 1 & 0 & t_2 \\ 0 & 0 & 1 & t_3 \\ 0 & 0 & 0 & 1 \end{pmatrix} \tag{10}$$

The ICP algorithm is initialised by setting the rotation and translation matrices equal to the identity matrix $\mathbf{R}=\mathbf{T}=\mathbf{I}$ and begins by identifying for each point $\vec{u}\in U$ the best match with the shortest distance $d(\vec{u},V)=min_{\vec{v}\in V}\|\vec{v}-\vec{u}\|$. This step can be efficiently performed using a k-d tree (Rusinkiewicz and Levoy, 2001). With the set of matching pairs as input, the best registration is calculated using the quaternion-based least square method, determining $\mathbf{R}$ and $\mathbf{T}$ which are then applied to U. The whole process is repeated iteratively, finding new matching points and their transformation, until the change in mean squared error falls below a given threshold.

The ICP algorithm, as described above, is used to find the best alignment of the stored shape to the evolving interface. This leaves each point within the interface in one of two possible states: it is either outside or inside of its aligned region. Let $S=\{S_1..S_n\}$ be the enclosed areas of each aligned shape to its corresponding level set function, $L=\{\Phi_1..\Phi_n\}$ be the set of level set functions at time t and $L^*=\{\Phi_1^*..\Phi_n^*\}$ the set of their temporary states, then

the final value of the level set function Φ_i^{t+1} at time step $t+1$ and position p is updated accordingly

$$\Phi_i^{t+1} = \begin{cases} \Phi_i^* & \text{if } (p \in S_i) \wedge (p \notin \{S \backslash S_i\}) \\ \max\left(\Phi_i^*, -\{L_j | p \in S_j\}\right) & \text{if } (p \in S_i) \wedge (p \cap \{S \backslash S_i\} \neq \emptyset) \\ \max\left(\Phi_i^*, -\{L^* \backslash \Phi_i^*\}\right) & \text{if } (p \cap S_i = \emptyset) \end{cases} \tag{11}$$

References

Paul J. Besl and Neil D. McKay. A method for registration of 3-D shapes. *Pattern Analysis and Machine Intelligence, IEEE Transactions on*, 14(2): 239–256, 1992. ISSN 0162-8828. doi: 10.1109/34.121791.

Jianhua Lin. Divergence measures based on the shannon entropy. *Information Theory, IEEE Transactions on*, 37(1):145–151, 1991. ISSN 0018-9448. doi: 10.1109/18.61115.

Szymon Rusinkiewicz and Marc Levoy. Efficient variants of the ICP algorithm. In *3-D Digital Imaging and Modeling, 2001. Proceedings. Third International Conference on*, pages 145–152, 2001. doi: 10.1109/IM.2001.924423.

James A. Sethian. *Level Set Methods and Fast Marching Methods: Evolving Interfaces in Computational Geometry, Fluid Mechanics, Computer Vision, and Materials Science ... on Applied and Computational Mathematics*. Cambridge University Press, 2nd edition, 1999. ISBN 0521645573.
